# MoRFchibi SYSTEM: software tools for the identification of MoRFs in protein sequences

**DOI:** 10.1093/nar/gkw409

**Published:** 2016-05-12

**Authors:** Nawar Malhis, Matthew Jacobson, Jörg Gsponer

**Affiliations:** 1 Michael Smith Laboratories—Centre for High-Throughput Biology, The University of British Columbia, Vancouver, BC V6T 1Z4, Canada; 2 Department of Biochemistry and Molecular Biology, University of British Columbia, Vancouver, BC V6T 1Z3, Canada

## Abstract

Molecular recognition features, MoRFs, are short segments within longer disordered protein regions that bind to globular protein domains in a process known as disorder-to-order transition. MoRFs have been found to play a significant role in signaling and regulatory processes in cells. High-confidence computational identification of MoRFs remains an important challenge. In this work, we introduce MoRFchibi SYSTEM that contains three MoRF predictors: MoRF_CHiBi_, a basic predictor best suited as a component in other applications, MoRF_CHiBi__*_Light_*, ideal for high-throughput predictions and MoRF_CHiBi__*_Web_*, slower than the other two but best for high accuracy predictions. Results show that MoRFchibi SYSTEM provides more than double the precision of other predictors. MoRFchibi SYSTEM is available in three different forms: as HTML web server, RESTful web server and downloadable software at: http://www.chibi.ubc.ca/faculty/joerg-gsponer/gsponer-lab/software/morf_chibi/

## INTRODUCTION

Protein–protein interactions (PPIs) play essential rolls in most biological processes in cells. Work in the last two decades has revealed that intrinsically disordered protein regions (IDRs) mediate many interactions as their structural flexibility enables them to ideally fit their target domain's binding surfaces (1). Currently, IDR binding sites are classified under two overlapping categories: short linear motifs (SLiMs) (2) and molecular recognition features or elements (MoRFs) (3). SLiMs are defined as conserved, short (3–10 amino acids) linear motifs that can mediate PPIs and other types of interactions (2). Importantly, SLiMs are not only found in IDRs, about 20% of known SLiMs are located in globular protein domains (2). MoRFs, on the other hand, are strictly located within IDRs. Additionally, MoRFs undergo disorder-to-order transitions upon binding to partners (3–7). Based on the structure they adopt upon binding, MoRFs are sub-categorized into three basic groups: α-MoRFs (form α-helices upon binding), β-MoRFs (form β-strands) and ι-MoRFs (form irregular structures) (8). While most MoRFs are shorter than 25 residues, some MoRFs are 50 or more residues long. MoRFs are found in proteins that are involved in diverse cellular processes in all three domains of life (8).

High accuracy computational identification of MoRFs remains a significant challenge in computational biology. A number of MoRF identification tools are currently available including ANCHOR (9), MoRFpred (10), fMoRFpred (8), MFSPSSMpred (11), DISOPRED3 (12), MoRF_CHiBi_ (13) and MoRF_CHiBi__*_Web_* (14). ANCHOR predicts MoRFs by estimating interaction energies between residues. MoRFpred and fMoRFpred utilize SVM models (and multiple sequence alignment for MoRFpred) in their predictions. MFSPSSMpred and DISOPRED3 predict MoRFs based on a SVM model with RBF kernel. MoRF_CHiBi_ utilizes two SVM models with sigmoid and RBF kernels to predict MoRFs relying on local physiochemical sequence properties. MoRF_CHiBi__*_Web_* predictions are generated by hierarchically incorporating scores of MoRF_CHiBi_ with those of IDR predictions and conservation assessments using Bayes rule. While the prediction precisions of the first five general MoRF predictors are about equal, MoRF_CHiBi__*_Web_* provides more than twice that precision. Other tools only target categories of MoRFs, including α-MoRF-Pred-I (15) and α-MoRF-Pred-II (16) that identify α-MoRFs, and retro-MoRF (17) that targets MoRFs with high sequence similarity to already known MoRFs or their reversed sequences. Furthermore, the recently developed DisoRDPbind method has an extended target space that covers intrinsically disordered regions involved in interactions with any type of partner including protein, RNA or DNA (18).

In this work, we introduce MoRFchibi SYSTEM, a series of MoRF predictors that serve different purposes and users. MoRFchibi SYSTEM includes these predictors in three forms: as HTML server, RESTful web server and downloadable software.

## MATERIALS AND METHODS

### Method

**Figure 1.**
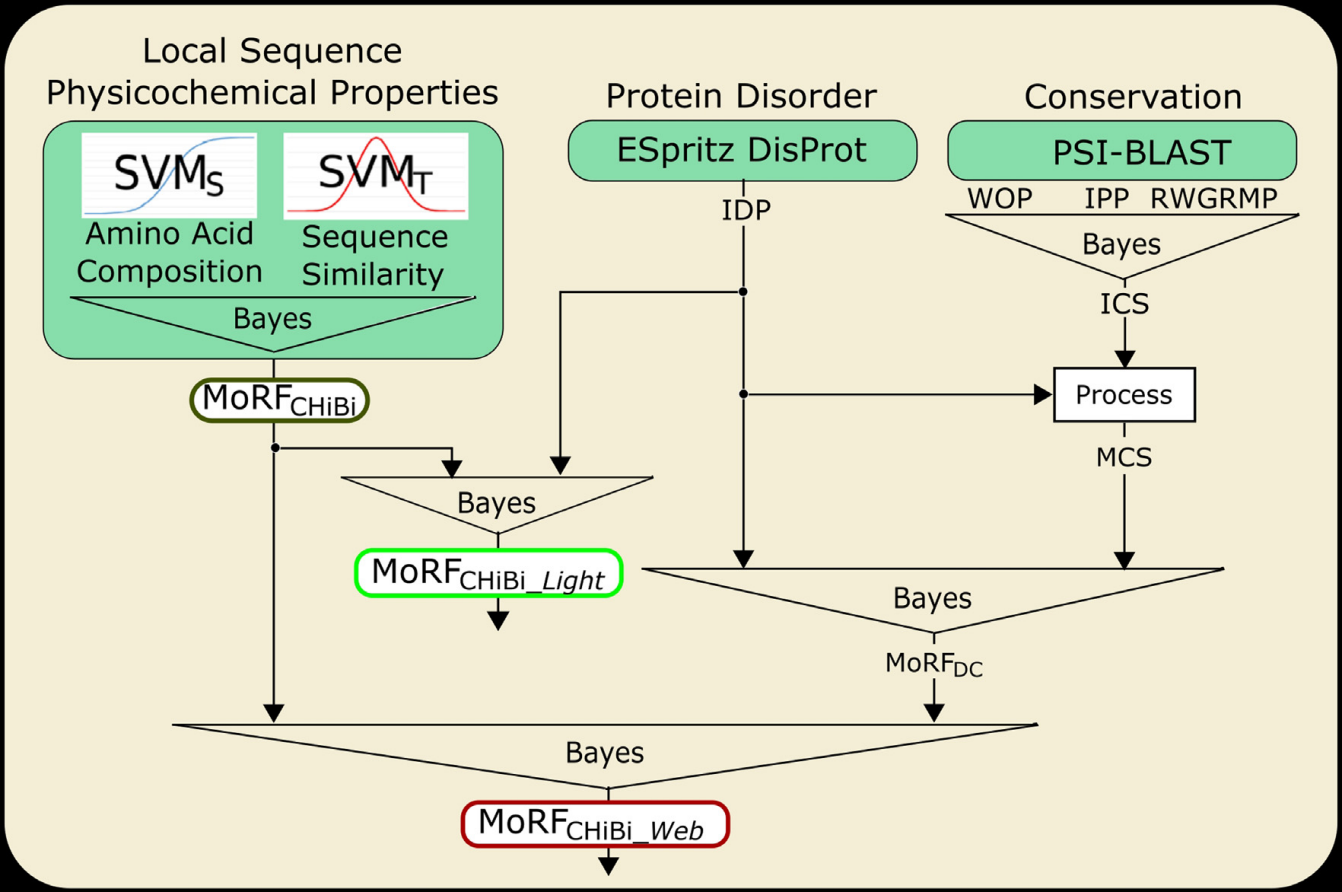
MoRFchibi SYSTEM. MoRFchibi SYSTEM contains predictors that use and integrate different data sources.

MoRFchibi SYSTEM includes three separate MoRF predictors; MoRF_CHiBi_, MoRF_CHiBi__*_Light_* and MoRF_CHiBi__*_Web_* (Figure 1).

MoRF_CHiBi_ relies on two SVMs modules to predict MoRFs based solely on local physicochemical sequence properties. MoRF_CHiBi_ is the least accurate choice in MoRFchibi SYSTEM. It processes more than 11 000 residues per minute (please see the benchmarking section and (13)).

MoRF_CHiBi__*_Light_* utilizes Bayes rule to incorporate MoRF_CHiBi_ scores with disorder scores generated by ESpritz (19). MoRF_CHiBi__*_Light_* is significantly more accurate than MoRF_CHiBi_ and it is the most accurate in targeting longer MoRF sequences among MoRFchibi SYSTEM predictors (MoRFs with more than 30 residues, see the ‘Benchmarking’ section). MoRF_CHiBi__*_Light_* processes more than 10 500 residues per minute.

MoRF_CHiBi__*_Web_* predictions are the most accurate in the MoRFchibi SYSTEM (please see the ‘Benchmarking’ section). They are generated by supplementing MoRF_CHiBi_ with disorder and conservation information. As functional elements, MoRFs are more conserved compared to other parts of IDRs (20, 21). Therefore, an initial conservation score (ICS) is assembled by incorporating three values from the PSI-BLAST (22) position specific scoring matrixes (PSSMs) using Bayes rule. Then, a MoRF conservation score (MCS) is obtained by processing ICS with intrinsic disorder predictions (IDP) (14). MoRF_DC_ is then computed by combining the MCS and intrinsic disorder predictions using Bayes rule. And finally, Bayes rule is used again to generate MoRF_CHiBi__*_Web_* from MoRF_DC_ and MoRF_CHiBi_. MoRF_CHiBi__*_Web_* processes ∼500 residues per minute.

### Datasets

One major challenge in the development of MoRF predictors is the sparseness of experimentally verified MoRFs that can be used for training and testing. To overcome this problem, the authors of MoRFpred (10) implemented an approach similar to that introduced by Mohan *et al*. (3), who searched the Protein Data Bank (23) for short peptides (potential MoRFs) that are in complex with longer protein partners (presumably globular domains). Disfani *et al*. (10) collected 885 sequences, each annotated by a single 6–25 residue long MoRF, and divided these sequences into a training set, TRAINING_HT and a test set, TEST_HT, such that sequences in TRAINING_HT share <30% identity with those in TEST_HT. TRAINING_HT, contains 421 sequences with 245 984 residues, 5396 of them in MoRFs and TEST_HT, contains 464 sequences with 296 362 residues, 5779 of them in MoRFs. (_HT; for high-throughput collection).

Although the large number of sequences in TEST_HT provides more robustness in the evaluation, this set is not ideal because most of its MoRFs are not experimentally validated to be disordered in isolation, it includes many homologous sequences (redundant), and each sequence is only annotated by a single MoRF (under annotated). Therefore, we assembled a second test set, TEST_EXP53. First, we joined four test sets that have previously been collected by the authors of ANCHOR (9), MoRFpred (10) and DISOPRED3 (12). MoRFs in these sets have been experimentally validated for their disordered character in isolation. Then we filtered out sequences with more than 30% identity to TRAINING_HT, as well as redundant sequences at a 30% identity cut-off. TEST_EXP53 has 53 sequences with a total of 2432 MoRF residues that we further divided into 729 from short MoRF sections (up to 30 residues) and 1703 from long MoRF sections (more than 30 residues). Importantly, in contrast to TEST_HT where each sequence is annotated by a single MoRF even if more may be present, sequences in TEST_EXP53 are annotated with all known MoRFs.

We also used a third test set, TEST_EXP9, to compare the prediction quality of the MoRFchibi SYSTEM predictors with that of MFSPSSMpred and DISOPRED3. These two SVM-RBF predictors are trained on an extended set of MoRFs including most of those found in our TEST_HT and TEST_EXP53 sets. The nine sequences of TEST_EXP9, collected by the authors of DISOPRED3, are not homologous to any sequence used in the training of DISOPRED3, MFSPSSMpred and the predictors of MoRFchibi SYSTEM. MoRFs in TEST_EXP9 have been experimentally validated to be disordered in the unbound state. TEST_EXP9 includes 12 MoRFs with 163 MoRF residues.

## BENCHMARKING

In the following, we will first summarize the comparison between the predictions made with MoRFchibi SYSTEM and other available servers. Details of this comparison can be found in Malhis *et al*. (14). Then, we will provide recommendations for the user of MoRFchibi SYSTEM based on results from this comparison.

Using TEST_HT and TEST_EXP53, we evaluated MoRFchibi SYSTEM predictions and compared them with those made by the most frequently used MoRF predictors in the field, MoRFpred, fMoRFpred and ANCHOR (Tables 1–3). Then, we used the much smaller TEST_EXP9 set to compare performances with those of MFSPSSMpred and DISOPRED3 (Table 4). We compared the area under the curve (AUC, in Table 1), the prediction specificity at given sensitivities (Tables 2 and 4) and the precision as a function of different sensitivities (Table 3).

**Table 1.** AUC results

Dataset	MoRF_CHiBi__*_Web_*	MoRF_CHiBi__*_Light_*	MoRF_CHiBi_	fMoRFpred	MoRFpred	ANCHOR
TEST_EXP	0.894, 0.755	0.868, 0.770	0.790, 0.679	0.662, 0.655	0.673, 0.598	0.683, 0.586
TEST_HT	0.806	0.777	0.743	0.646	0.675	0.605

AUC values of MoRFchibi SYSTEM predictors compared to those of fMoRFpred, MoRFpred, and ANCHOR using TEST_EXP53 and TEST_HT. We evaluated MoRF predictions for short MoRFs (up to 30 residues) separately from long MoRFs (more than 30 residues). Thus, AUC results for the TEST_EXP53 set are in the form: short, long.

**Table 2.** Specificity as a function of sensitivity

	Specificity (short, long)					
Sensitivity	MoRF_CHiBi__*_Web_*	MoRF_CHiBi__*_Light_*	MoRF_CHiBi_	fMoRFpred	MoRFpred	ANCHOR
0.2	0.990, 0.980	0.987, 0.983	0.989, 0.961	0.947, 0.924	0.941, 0.901	0.930, 0.872
0.4	0.968, 0.911	0.952, 0.914	0.935, 0.834	0.816, 0.803	0.846, 0.748	0.825, 0.690

Specificity as a function of sensitivity computed on the TEST_EXP53 set (short, long) for MoRF_CHiBi_*Web*_, MoRF_CHiBi_*Light*_, and MoRF_CHiBi_ compared to that of fMoRFpred, MoRFpred, and ANCHOR.

**Table 3.** Precision as a function of sensitivity

	Precision [Naïve precisions are (0.031, 0.070)]					
Sensitivity	MoRF_CHiBi__*_Web_*	MoRF_CHiBi__*_Light_*	MoRF_CHiBi_	fMoRFpred	MoRFpred	ANCHOR
0.2	0.40, 0.44	0.34, 0.47	0.39, 0.28	0.11, 0.16	0.10, 0.13	0.08, 0.10
0.4	0.29, 0.25	0.21, 0.26	0.16, 0.15	0.06, 0.13	0.08, 0.11	0.07, 0.09

Precision as a function of sensitivity computed on the TEST_EXP53 set (short, long) for MoRF_CHiBi_*Web*_, MoRF_CHiBi_*Light*_, and MoRF_CHiBi_, compared to fMoRFpred, MoRFpred, and ANCHOR.

**Table 4.** Comparing with MFSPSSM and DISOPRED3

	Specificity				
Sensitivity	MoRF_CHiBi__*_Web_*	MoRF_CHiBi__*_Light_*	MoRF_CHiBi_	MFSPSSMpred	DISOPRED3
0.147	0.990	0.993	0.996		0.958
0.206	0.988	0.989	0.980	0.900	

Specificity as a function of sensitivity computed on the TEST_EXP9 set for MoRF_CHiBi_*Web*_, MoRF_CHiBi_*Light*_, and MoRF_CHiBi_ compared to MFSPSSMpred and DISOPRED3. Sensitivity and specificity values for the latter two predictors were taken from Jones and Cozzetto (12).

These comparisons reveal that all three MoRFchibi SYSTEM predictors perform better than other methods regardless of which evaluation metric is used. Importantly, MoRF_CHiBi__*_Web_* generated less than half the false positive rate for the same true positive rate at any practical threshold values (see (14)). The comparison (Tables 1–3) also reveals that MoRFchibi SYSTEM predictors, MoRFpred, fMoRFpred and ANCHOR identify short MoRFs better than long ones. This may be expected as all these predictors were trained on datasets that contain only short MoRFs. The results on TEST_EXP53 further reveal a limited contribution of conservation information to the identification of long MoRFs. MoRF_CHiBi__*_Web_*, which uses conservation information, does not perform as well in the identification of long MoRFs as MoRF_CHiBi__*_Light_*, which may suggest that the percentage of conserved residues in long MoRFs is lower than that in short MoRFs.

For MoRF predictors that are based on machine learning, the problem of over scoring MoRFs that are very similar to those used in its training can lead to novel MoRFs being masked by those over scored training MoRFs. With only one of the four sub-components of MoRF_CHiBi__*_Web_* directly trained on its training data (13), MoRF_CHiBi__*_Web_* provides high scoring consistency compared to single module predictors. To measure this consistency, we compared the MoRF_CHiBi__*_Web_* performance on its training set TRAINING_HT to that on the TEST_HT. Results show only a small difference in MoRF_CHiBi__*_Web_* performances between the two sets (an AUC of 0.825 for TRAINING_HT versus 0.806 for TEST_HT).

Based on these results and the processing speeds (see above) of the different MoRFchibi SYSTEM predictors, the following recommendations for users can be made:

MoRF_CHiBi__*_Web_* is the most accurate in MoRFchibi SYSTEM and outperforms previously developed predictors significantly (significance assessed with t-Test; all *P*-values are available on the server's webpage). However, it is rather slow because the calculation of conservation scores requires a time consuming multiple sequence alignment step. Thus it is most appropriate for low-throughput, high-accuracy MoRF predictions. It is particularly strong in the search for short (<30 residues) MoRFs.

MoRF_CHiBi__*_Light_* is not far behind MoRF_CHiBi__*_Web_* in terms of its prediction performance. However, it is much faster and, therefore, most appropriate for high-throughput MoRF predictions. It shows a small advantage over MoRF_CHiBi__*_Web_* in the search for long (>30) MoRFs (Tables 1–3).

MoRF_CHiBi_, is the least accurate among the three MoRFchibi SYSTEM predictors but still superior to the other available predictors. As its predictions are solely based on information learned from a training set of MoRFs, it is least likely to interfere with other parts when integrated into multi-unit bioinformatics tools. It is also the fastest in MoRFchibi SYSTEM.

## SERVER DESCRIPTION

### Input

The input for MoRFchibi SYSTEM is the primary amino acid sequence in fasta format. To balance priorities of different users, requests to the HTML and the RESTful web servers are limited to a single sequence each. However, there is no limit on the number of sequences that can be processed in each run of the downloadable software.

### Output

The output is presented in two different forms: a downloadable text table and an interactive graphic chart. Six propensity scores are generated for each residue in the query sequence:

- The three MoRFchibi SYSTEM predictions: MoRF_CHiBi__*_Web_* (MCW), MoRF_CHiBi__*_Light_* (MCL) and MoRF_CHiBi_ (MC).
- The intrinsic disorder prediction (IDP) based on ESpritz.
- The initial conservation propensity score (ICS) (14).
- Another MoRF prediction MoRF_DC_ (MDC) that is based on the combination of the disorder prediction and the ICS (see ‘Method’ section and (14)).

Each of these scores is normalized to approximately fit a Gaussian probability density function specified by the normal distribution N(0.5, 0.01) and is limited to the range [0..1] as described in the article (14). In addition, the downloadable release includes two high-throughput options, one only generates the MC scores, and the other generates the MC and the MCL scores.

### Usage example

**Figure 2.**
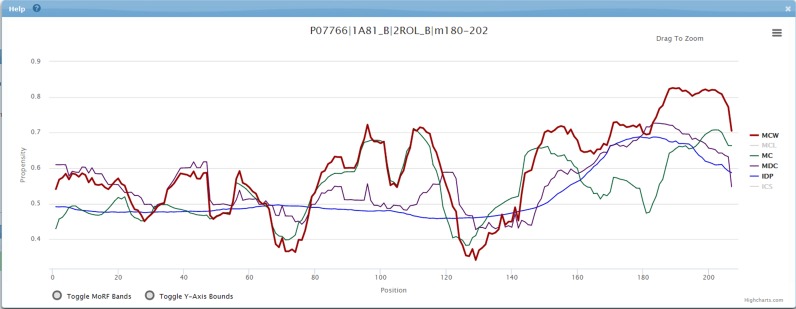
Graph from the HTML server for the predictions of the CD3E_HUMAN protein. The propensity scores provided by MoRF_CHiBi__*_Web_* (MCW), MoRF_CHiBi_ (MC), MoRF_DC_ (MDC) and the intrinsic disorder protein prediction (IDP) are shown in red, green, purple and blue, respectively. MoRF_CHiBi__*_Light_* (MCL) and conservation (ICS) are switched off.

The CD3E human protein P07766 has a disordered region at its C-terminus (residues 153–207) (24). This IDR includes a MoRF that covers residues 180–202 (PDB: 1A81_B and PDB: 2ROL_B). MoRF propensity scores generated by MoRF_CHiBi_ (MC), which are based on the local physicochemical properties of the sequence, correctly identify this MoRF region (Figure 2, green curve). However, MoRF_CHiBi_ scores for residues 80–117 and 142–164 are similarly high. Combining disorder predictions and conservation information in the MoRF_DC_ (MDC, purple curve) provides high prediction scores for the region 170–200, which is longer than the actual MoRF. The integration of the MoRF_CHiBi_ and MoRF_DC_ scores in MoRF_CHiBi__*_Web_* (MCW, red curve) provides the best result with a clearly distinct peak in the score chart between residues 180–202, which is where the MoRF is located.

### The CHiBi server overview

**Figure 3.**
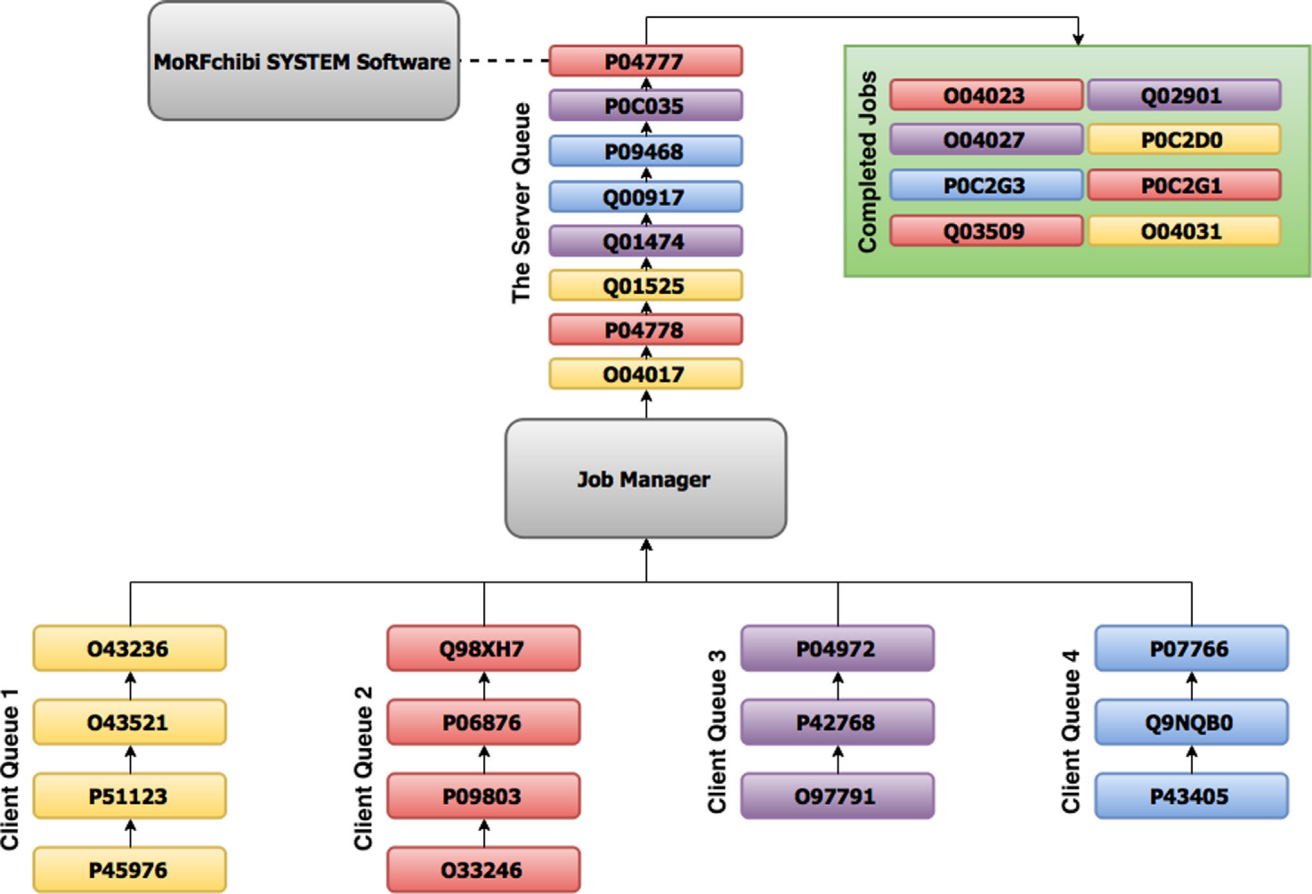
An example for the MoRFchibi SYSTEM two tiers queue structure. Eight jobs are in the server queue, two from each client. The ‘red’ client's job at the top of the server queue [P04777] is being processed by the MoRFchibi SYSTEM software. Jobs in the ‘completed Jobs’ section (top right) can be accessed through their associated URL links. Once [P04777] is completed, it will be released from the server queue and the job at the top of the ‘red’ client queue [Q98XH7] will be moved by the Job Manager to the tail of the server queue.

Once a query sequence is submitted to either the HTML or the RESTful web servers, a job object is created and a URL address pointing to its future results is returned to the client. To prevent being dominated by a large number of query sequences from a single ‘client’ (defined below), each server utilizes a two tiers queue structure (Figure 3). Jobs are inserted into the first-in first-out server queue while the job at the top of the queue is been processed by the MoRFchibi SYSTEM software. Each client can place up to two jobs in the server queue, if more sequences are submitted by a single client, extra jobs are placed temporarily in that client's private queue. Once a client job at the top of the server queue is completed, it will be released from the queue and the job at the top of that client queue (when exist) will be moved by the job manager to the tail of the server queue. Client queues are located on the server, thus, once the links to the future result pages are secured, users can safely disconnect from the server.

Two main differences exist between the HTML and the RESTful servers: first, clients in the HTML server are browser sessions, and they are IP addresses in the RESTful web server. Second, in the HTML server, client queues are not limited in size, and they are limited to 200 jobs in the RESTful web server.

## FINAL REMARKS

In this paper, we introduced MoRFchibi SYSTEM, a set of software tools for identifying MoRF locations in amino acid sequences. MoRFchibi SYSTEM includes three predictors: MoRF_CHiBi_ which is best suited as a component predictor in other applications, MoRF_CHiBi__*_Light_*, which was built to process large datasets and MoRF_CHiBi__*_Web_* which is best suited for high accuracy predictions in small and medium size datasets. In addition, MoRF_CHiBi__*_Web_* provides scoring consistency so that novel MoRFs are not overshadowed by those used in its training. MoRFchibi SYSTEM is available in three forms: a HTML server, a RESTful web server and a downloadable software. Compared to the beta versions associated with (13) and (14), this full release of MoRFchibi SYSTEM includes MoRF_CHiBi__*_Light_*, a RESTful web server with a template interface code in Python and a downloadable software package with all three MoRFchibi SYSTEM predictors. MoRFchibi SYSTEM is fully documented, including a tutorial video that covers the principles of its three predictors. Furthermore, a number of extra features are added (see the overview on the server's webpage) and most of the original C++ code has been rewritten in order to increase the processing speed, e.g. the new MoRF_CHiBi_ provided here has about twice the processing speed of the beta version associated with (13).
